# Virtual Reality and Stress Management: A Systematic Review

**DOI:** 10.7759/cureus.64573

**Published:** 2024-07-15

**Authors:** Shakila Meshkat, Mahsa Edalatkhah, Corinna Di Luciano, Josh Martin, Gursharanjit Kaur, Gyu Hee Lee, Haley Park, Andrei Torres, Ali Mazalek, Bill Kapralos, Adam Dubrowski, Venkat Bhat

**Affiliations:** 1 Interventional Psychiatry Program, St. Michael’s Hospital, Toronto, CAN; 2 Department of Psychology, University of Waterloo, Waterloo, CAN; 3 maxSIMhealth Laboratory, Faculty of Health Sciences, Ontario Tech University, Toronto, CAN; 4 Synaesthetic Media Lab, Toronto Metropolitan University, Toronto, CAN; 5 maxSIMhealth Group, Ontario Tech University, Oshawa, CAN; 6 Health Sciences, Ontario Tech University, Montreal, CAN; 7 Psychiatry, University of Toronto/St. Michael's Hospital, Toronto, CAN

**Keywords:** relaxation, systematic review, stress, stress management, vr, virtual reality

## Abstract

Amidst the growing prevalence of chronic stress and its potential negative impacts on mental health, this review explores the use of virtual reality (VR) as a stress management solution, aiming to assess its viability and effectiveness in this context. A comprehensive search was conducted on MEDLINE, PsycINFO, and Embase from inception until February 2024. Eligible studies were primary research papers that focused on the use of VR as an intervention to mitigate psychological stress and/or distress. We included studies where the assessment of stress levels primarily relied on self-report measures. A total of 50 studies involving 2885 participants were included in our systematic review. VR-based interventions varied across studies, implementing tools such as cognitive behavioural therapy, exposure therapy, mindfulness and relaxation, repetition tasks, and psychoeducation. The reviewed studies yielded mixed results; however, a strong indication was present in highlighting the promising potential of VR-based interventions. Many studies observed a decrease in psychiatric symptoms in participants and reported increased quality of life. Various studies also found VR to be a valuable tool in promoting stress reduction and relaxation. VR was proven useful in exposing participants to stressors in a safe, controlled way. These potential benefits appear to come with no risk of harm to the participants. Although the findings are heterogenous, there is sufficient evidence supporting the use of VR for stress management across a range of contexts and populations. Overall, VR appears to be a generally low-risk, feasible intervention for those struggling with stress.

## Introduction and background

In our rapidly evolving modern society, the issue of chronic stress has risen to a significant prominence. Persistent stress poses a significant challenge in our modern, fast-paced society. The constant influx of information urging us to take action can make it increasingly difficult to quiet our minds [1]. When faced with environmental stressors, our body responds with physiological changes, such as an increased heart rate due to heightened activity in the sympathetic nervous system [2]. After the stressor subsides, the parasympathetic branch of the autonomic nervous system takes over, restoring balance to the body [3]. While this mechanism is beneficial when exiting a stressful situation, it becomes problematic in our current reality where individuals frequently encounter stressors, resulting in a chronic state of imbalance [2,3]. The stress-vulnerability model proposes that the outcome of a stressful situation, whether it remains within a manageable threshold or leads to mental health challenges, hinges on the intricate interplay between the experienced stress level and an individual's capacity to cope with it-commonly referred to as their vulnerability [4]. It is noteworthy that while stress is frequently linked with various psychiatric disorders, including but not limited to anxiety disorders, depressive disorders, and post-traumatic stress disorder (PTSD), the relationship between stress and these conditions is complex [5].

Stress often exacerbates or triggers symptoms in individuals predisposed to such disorders due to a complex interplay of genetic, environmental, and psychological factors [6]. Research indicates a potential involvement of the hypothalamic-pituitary-adrenal (HPA) axis in the genesis of major psychiatric conditions such as depression, mania, psychosis, and anxiety disorders [7]. Elevated stress levels prompt an upsurge in cortisol production, and persistent hypercortisolemia contributes to the development of glucocorticoid receptor tolerance [8]. This alteration further impacts the hippocampus, a brain region abundant in corticosteroid receptors [9]. Perturbations in hippocampal function can lead to inappropriate emotional responses, and variations in hippocampal volume have been observed in diverse psychiatric disorders, including schizophrenia, PTSD, borderline personality disorder, and depression [10,11].

Understanding the intricate interplay between stress, fear, anxiety, and other related constructs is essential in comprehending the complexities of human emotional and physiological responses. Stress, often triggered by external pressures or internal challenges, can evoke fear and anxiety as adaptive responses aimed at coping with perceived threats [12]. Fear arises in response to immediate danger, activating the body's fight-or-flight response, while anxiety manifests as apprehension or worry about potential future threats [13]. These constructs share common neural circuits and physiological pathways, including the amygdala and the HPA axis, illustrating their interconnected nature [12-14]. Moreover, chronic stress and anxiety can exacerbate each other, leading to detrimental effects on mental health and overall well-being [14].

The American Psychological Association reports that 76% of surveyed US adults are negatively impacted in some way by the stress they experience. It was also reported that 27% of the surveyed adults stated they are unable to function due to the stress they experience [15]. According to the UK National Institute for Health and Clinical Excellence (NICE), enhancing mental health management at work, which involves stress prevention, early intervention, and problem identification, could potentially reduce productivity losses by up to 30% and lead to annual savings of £250,607 in a company with 1000 employees [16]. Therefore, the imperative for effective stress treatments has never been more pressing. Stress, often triggered by factors ranging from work-related pressures to personal challenges and societal expectations, exerts profound adverse effects on both mental and physical well-being [17]. Chronic stress is closely linked to an array of conditions, including psychiatric disorders, cardiovascular diseases, and compromised immune systems [5,18]. Unearthing efficacious stress management strategies, therapies, and interventions is paramount to enhance individuals' holistic wellness, elevate productivity, and alleviate the strain on healthcare systems. By devising approaches that directly target stress management, we have the potential to empower individuals to lead lives marked by improved health and greater contentment, thereby nurturing a society characterized by enhanced resilience.

Virtual reality (VR) has emerged as a captivating solution for stress management, offering a unique avenue to unwind and find respite from the demands of modern life [19]. VR has also proven to be effective in alleviating symptoms and improving outcomes for individuals dealing with both psychiatric conditions such as PTSD and various health conditions including pain management [20,21]. By immersing users in visually stunning and emotionally engaging environments, VR provides a sensory escape that can effectively lower stress levels and promote relaxation [22]. From serene nature scenes to guided mindfulness exercises, VR offers a versatile range of experiences tailored to individual preferences [19]. The recognition of stress's pervasive influence underscores the imperative for comprehensive stress management strategies. However, the multifaceted nature of stress, ranging from daily stressors to those associated with psychiatric disorders, necessitates a nuanced approach to its mitigation. To address this complexity, this review seeks to explore various forms of stress and their related management within a unified framework, particularly focusing on the role of VR. Despite its potential, a significant gap in knowledge exists regarding the sustained impact of VR on stress management. As VR continues to integrate into wellness practices and stress-related disorders continue to rise in prevalence, further research and exploration are crucial to fully understand its role in providing effective and sustainable stress relief. The objective of this review is to systematically synthesize the current literature to determine the efficacy and viability of VR in stress management. The objective of this review is to systematically synthesize the current literature to determine the efficacy and viability of VR in treating stress and related disorders.

## Review

Method

Search Strategy

A comprehensive search was conducted on MEDLINE, PsycINFO, and Embase through OVID from inception until February 23, 2024. This systematic review was completed based on the Preferred Reporting Items for Systematic Review and Meta-Analysis (PRISMA) Statement [23]. We used the following keywords [Mesh] to identify relevant articles: (Stress*, Psychological) OR (life OR psychologic* OR emotion* OR Psychological Distress OR Rehabilitation OR Treatment* OR Intervention* OR Prevention) AND virtual reality OR VR. There was no restriction on the year of publication. Google Scholar and included studies' references were evaluated to avoid missing relevant articles.

Study Eligibility Criteria

All articles were imported to the Covidence platform (covidence.org) for the screening process. Two reviewers independently evaluated titles, abstracts, and full-text articles from the electronic search based on predefined inclusion and exclusion criteria to pinpoint potentially eligible articles. Any disagreements between the two reviewers were resolved through discussion. Studies eligible for the purposes of this review were primary research papers in the English language that focused on the use of VR as an intervention to mitigate psychological stress and/or distress. In the context of this study, VR was defined as a technology that immerses users in computer-generated environments through various implementations. These implementations encompass head-mounted displays (HMDs) for immersive visual experiences, simulators for professional training, mobile VR for accessibility and web-based VR for browser-driven experiences. In contrast, web-based VR for browser-driven experiences involves interactive environments accessed through web browsers, allowing users to navigate and interact with virtual content in real-time, while 360° videos are prerecorded videos capturing panoramic scenes that users can view from any angle but do not offer interactive elements. Study populations included healthy and clinical samples of adults and older adults (aged 18+). Eligible studies must also have incorporated a measure of psychological stress and/or distress before and after the VR intervention with the use of 1) formally recognized assessment tools (e.g., perceived stress scale (PSS)); or 2) self-report; or 3) clinical assessments. Studies that use physiological measurements (e.g., heart rate variability and skin conductance) as the only measure of stress were excluded. Our rationale for primarily including studies that relied on self-report measures stems from the nature of the interventions evaluated in our review. Given the immersive and experiential nature of VR interventions, self-report measures are often considered the most appropriate and practical method for assessing stress levels and psychological distress experienced by participants during VR sessions. While physiological measures are valuable in stress research, their incorporation into VR interventions is less common and may not align with the specific focus of our review. We also excluded letters/correspondences, animal studies, editorials, systematic reviews, meta-analyses, commentary, case studies, case series, observational studies and conference abstracts.

Data Extraction

Two reviewers independently read the full text of eligible studies and extracted the following data: author, year of publication, country, apparatus, outcome measure, study design, topic results, participants, and conclusion.

Risk of Bias Assessment

We employed the Cochrane Handbook for Systematic Reviews of Interventions to assess the quality of eligible randomized trials. Our evaluation encompassed five domains: potential bias from random sequence generation, allocation concealment, blinding of study participants, incomplete outcome data, and selective reporting [24]. The Newcastle-Ottawa Scale (NOS) was used to assess potential bias in observational studies. The NOS allowed us to evaluate the quality of these studies based on three key domains: selection of study groups, comparability of groups, and assessment of outcome. This comprehensive approach enabled a thorough examination of bias and methodological rigour within the realm of observational research [25]. The risk of bias assessment of included studies is indicated in Supplementary materials.

Results

Search Results

**Figure 1:**
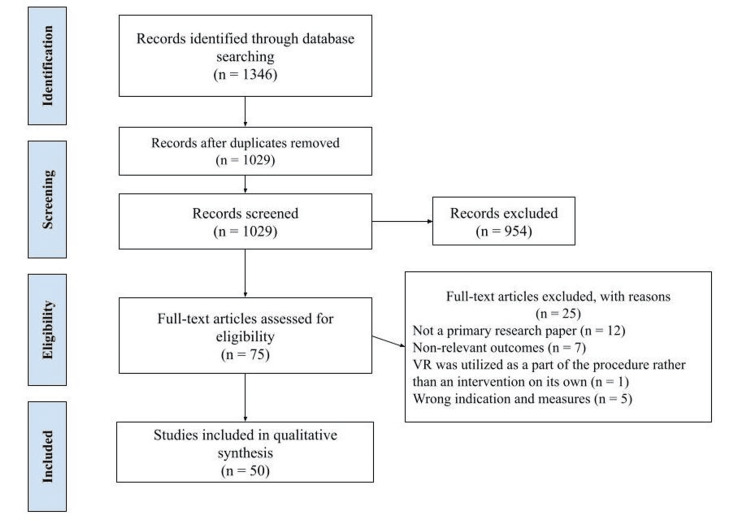
Preferred Reporting Items for Systematic Reviews and Meta-Analyses (PRISMA) flow diagram.

The study flow diagram is indicated in Figure 1. A total of 1346 studies were identified through database searching. After removing the duplicates (n = 317), two reviewers independently scanned the titles and abstracts of 1029 papers. The full texts of 75 articles were screened for eligibility. Twenty-five articles were excluded for the following reasons: not a primary research paper (n = 12), non-relevant outcomes (n = 7), VR was utilized as a part of the procedure rather than an intervention on its own (n = 1), wrong indication and measures (n = 5). Eventually, 50 studies met the inclusion criteria involving 2885 participants and were included in our systematic review. Risk of bias assessment results are indicated in Supplementary materials. The characteristics of included studies included in this review are summarized in Table 1.

**Table 1:** Characteristics of included studies. PROMIS: Patient-Reported Outcomes Measurement Information System, FAS: Facial Anxiety Scale, HC: heart coherence, PSYRATS: Psychotic Symptoms Rating Scale, FCOR : fear of coronavirus, SRSI3: Smith Relaxation State Inventory 3, SSQ: Simulator Sickness Questionnaire, MoCA : Montral Cognitive Assessment, DASS 21: Depression, Anxiety, and Stress Scale 21; PANSS: Positive and Negative Symptom Scale; SSI-4: stuttering severity instrument 4; GAD-7: Generalised Anxiety Disorder-7 item measure; SUDs: Subjective Units of Distress; PA: positive affect; NA: negative affect; LSAS: Liebowitz Social Anxiety Scale; HAM-d: Hamilton Rating Scale for Depression; VAS: visual analog scale; VAS-A: Visual Analogue Scale for Anxiety; STAI: State-Trait Anxiety Inventory Form; PSS: Perceived Stress Scale; MINI: Mini-International Neuropsychiatric Interview; SCL-90-R: Symptom Checklist 90-Revised; GSI: Global Severity Index; ASDS-2: Adapted Symptom Distress Scale-2; PFS: Piper Fatigue Scale; STAI: Spielberger Anxiety State Inventory; SCL: Skin Conductance Level; BHS: Beck Hopelessness Scale; HADS: Hospital Anxiety and Depression Scale; SCS: Social Connectedness Scale; IES-R: Impact of Event Scale-Revised; EQ-5D: European Quality of Life, 5 dimensions; SCID-5-CV: Structured Clinical Interview for DSM-5 Disorder; WIWI: Was it Worth it Questionnaire; FACT-B: Functional Assessment of Cancer Therapy-Breast cancer patient; DT: Distress Thermometer; SAS: Self-Rating Anxiety Scale; SDS: Self-Rating Depression Scale; DASS: Depression Anxiety Stress Scale; BHS: Beck Hopelessness Scale; FAS: Flight Anxiety Situations, B-MEPS: Brief measure of emotional preoperative stress; NVFAS: novel visual facial anxiety scale; NSRS: single-item numeric stress rating scale; PQ: presence questionnaire; CALM: Cancer And Living Meaningfully

Author	Country	Topic	Intervention	Apparatus	Study design	Participants	Outcome measure	Results	Conclusion
Reger et al. 2019 [26]	USA	VR and PTSD	10 sessions of prolonged exposure or 10 sessions of VR exposure therapy	The VR system for Iraq/Afghanistan includes a clinician's interface, a Dell XPS desktop, an eMgin z800 head-mounted display, a Logitech gaming joystick or mini joystick, digital microphones, over-the-ear headphones, an EnviroScent Scent Palette, a platform with bass shaker speakers.	RCT	Active-duty US Army soldiers who had developed PTSD during a military deployment N = 96; PE (n = 47); VRE (n = 49) PE: Mean age = 30.74 (SD = 6.97); VRE: Mean age = 29.76 (SD = 6.50)	SUDS CAPS	There were no significant differences in SUDS scores between the VRE and PE groups during in-session or imaginal exposure at Session 3 (3.65, 95% CI (-4.22, 11.52)) or Session 10 (-0.98 (95% CI = -14.84, 12.95)). Both groups showed a decrease in mean and peak SUDS, but this change was not statistically significant.	The study does not provide support for the hypothesis that VRE enhances emotional engagement with traumatic memories.
Norr et al. 2019 [27]	USA	VR and PTSD	10 weekly sessions of PE/VRE	The VR Iraq/Afghanistan system features a Dell XPS desktop, an eMagin z800 HMD with an inertia cube tracker, a Logitech or mini joystick attached to a mock M4 rifle, over-the-ear headphones, bass shaker speakers for vibration, and an EnviroScent Palette.	RCT	Active-duty soldiers with PTSD resulting from deployment-related trauma (N = 108)	CAPS SUDs	All symptom clusters decreased markedly over time (reexperiencing Cohen's d = −0.74, hyperarousal d = −1.44, avoidance/numbing d = −1.68). Decreases in in-vivo SUDs were associated with decreases in PTSD symptoms over the course of treatment (p<0.001). Changes in imaginal SUDs did not predict change in PTSD symptoms (p=0.859).	In-vivo exposures are possibly more closely tied to changes in overall PTSD symptoms than imaginal exposures during exposure therapy.
Loucks et al. 2019 [28]	USA	VR and PTSD	6-12 VRE sessions	The VR MST-specific stimulus content was created using the BRAVEMIND VR exposure system. During exposure, clinicians were able to customize the ambient settings and trigger stimuli in real-time via a separate interface.	RCT	Military veterans who experienced military sexual trauma during their time in service (N = 15, Age: Mean: 46, Range: 32-72 years)	CAPS-5; MINI PCL-5 PHQ-9 CTQ-SF	The study found significant reductions in CAPS (p=0.004), self-reported PTSD (p=0.022), and depressive symptoms (p=0.004) from pretreatment to post-treatment, with no significant changes at the 3-month follow-up. Effect sizes were larger post-treatment, and a considerable percentage of participants showed meaningful reductions in PCL-5 and PHQ-9 scores.	VR is a safe and feasible approach for treating MST-related PTSD, with significant reductions in clinician-assessed and self-reported PTSD and depressive symptoms from pretreatment to post-treatment, which were maintained at the 3-month follow-up.
Kim et al. 2022 [29]	South Korea	VR and social anxiety	VR self-training (three environments related to school, business and daily life) or the waiting list	HMD consisting of a Samsung Galaxy S6 latched onto Samsung Gear VR powered by Oculus	RCT	Volunteers who were evaluated as having high social anxiety through the screening process by a psychiatrist (N = 61 Age Range: 19-30)	HADS	The VRS group showed significant decreases in LSAS anxiety and avoidance scores, distress index, and negative self-evaluation index (p<0.001) in the follow-up assessment compared to the initial assessment. These changes were not observed in the WL group (p>0.05). Additionally, neural activity in the right lingual gyrus and the left thalamus significantly increased in the VRS group between the initial and follow-up scans.	These short-term neural changes support VRS as an initial intervention option for patients with severe social anxiety who may be reluctant to pursue formal treatment.
Freeman et al. 2016[30]	UK	VR and persecutory delusions	VR cognitive therapy, VR exposure	Head-mounted display linked to a computer and tracking system	RCT	Patients with persecutory delusions (N = 30 Threat belief testing group (n = 15); Exposure Group (n = 15) Mean age: 40 years)	PANSS PSYRATS, Safety Behaviours Questionnaire-Persecutory Beliefs, Beck Anxiety Inventory (BDI)	In comparison to VR exposure therapy, VR cognitive therapy resulted in a 22% decrease in delusional conviction (p = 0.024, Cohen's d = 1.3). In behaviour test ratings, VR Cognitive Therapy led to a 19.6% reduction in real-world distress compared to VR Exposure (p = 0.020; Cohen's d = 0.8).	Compared to VR exposure, VR cognitive therapy was more effective in reducing paranoid thinking.
Hoch et al. 2012[31]	USA	VR and its feasibility + stress	Virtual world technology with mind-body practitioners	Computer with speakers	Pilot trial	General population (N = 24 Age: Mean = 42 (SD = 13))	PSS SCL-90-R GSI Depression Scale (DEP)	Scores on the PSS and all three scales of the SCL90-R were normally distributed and there was a trend toward improvement. There was a general trend toward decreased prescribed stress (15.7 to 15.0), symptoms of depression (57.6 to 57.0), and anxiety (56.8 to 54.8). There was also a significant decrease of 2.8 points on the SCL-90-R GSI (p<0.05).	It is feasible to deliver a typical mind-body intervention through a virtual environment and it is well received; however, the user interface can be a challenge as recruitment was limited to participants with prior experience in SL.
Richesin et al. 2021[32]	USA	Art making, VR, and stress	1: Classic art-making intervention in 2-dimensions, 2: novel art-making intervention in 3-dimensional VR, 3: non-artistic control intervention in VR	HTC Vive headset; laptop computer; Oculus Quest headset; 2D: Crayola brand washable markers, coloured pencils, crayons, sharpie brand permanent markers and Ticonderoga brand #2 pencils; tall easel; Post-it easel pad	RCT	Undergraduate students (N control= 16, N 2D (i.e., classic art-making) = 13, N 3D (i.e., VR) = 16 Age: mean= 21.20, SD= 2.27))	PANAS STAI PSS Heart rate SC salivary alpha-amylase (sAA)	No significant group differences except for heart rate (p>0.05). All groups showed decreased HR, negative affect, state and trait anxiety over time. The 3D group had a more significant HR decrease compared to the control group. Other variables didn't show significant changes.	Art making, whether in two or three dimensions can reduce levels of stress and anxiety in a non-clinical population. VR office rooms can reduce measures of stress and anxiety.
Kothgassner et al. 2019 [33]	Austria	VR and stress, differences depending on the source of support	An avatar, an agent, a real human or received no support before completing the TSST and two subsequent social behaviour tasks	Head-mounted display with an externally attached head-tracking system	RCT	Ad-hoc sample of students (N=14 in each group Age: mean= 24.36, SD= 3.440)	HR, manipulation check, German Social Support Questionnaire (Networked Minds Measure of Social Presence (NMMSP) VAS)	Real and avatar support groups showed higher perceived social support compared to others. No significant differences in NMSQ scores (p>0.05). Both avatar and real support reduced worry and affected prosocial behaviour after a stress-inducing task, with no significant differences in heart rate between avatar and agent conditions (p>0.05).	Participants who had been emotionally supported by the confederate actor – either via an avatar or face-to-face – kept less seating distance from the confederate
Villani and Riva 2012[34]	Italy	VR and stress management	VR, video, audio	Photographs; wireless joystick; head-mounted display; Sony Glastron; Audiotape with headphones.	RCT	General population (N=12 in each of the three conditions range= 18-35, mean Men= 25,21, SD= 1.44, mean Women= 25.47, SD= 0.87)	Mesure du Stress Psychologyque (MSP) state-trait anxiety inventory heart rate	The ANOVA results on gain scores showed significant differences between video (M = -5.57, SD = 1.01) and VR (M = -3.80, SD = 1.41) groups (p < 0.001) and between video and audio (M = -4.83, SD = 1.49) groups (p = 0.006) in reducing state anxiety. In particular, VR reached a better HR reduction compared to audio and video groups. STAI: significant effect of time, and condition, but no interaction. Video conditions reached better results related to the anxiety state reduction in the guided sessions.	All three interactive experiences were effective in inducing positive emotions and integrating stress management approaches. VR, in particular, demonstrated superior results in terms of psycho-physiological changes.
Tan et al. 2021[35]	Singapore	VR and stress management programme for inpatients with mental disorders	Virtual screen-based stress management programme	iTV Goggles	RCT	Patients with mild or moderate mental disorders that affected their daily activities and social-occupational functioning (N= 41, N WL= 21, N intervention= 20 Age range 21-65)	PSS NSRS BP HR, Perceived Relaxation Scale Knowledge on Stress and Medication Management Questionnaire (KSMMQ)	The VR group had significantly lower NSRS score than the control for sessions 1 and 2 (p<0.05). Non-significant between-group differences on PSS (p>0.05). The VR group had significantly higher ST in Session 1, and non-significant differences in post-test scores of HR, systolic and diastolic BP for either Session. The VR group reported significantly higher perceived relaxation and knowledge improvement in Session 1 (p<0.05).	VR had a positive effect on knowledge and perceived relaxation
Aganov et al. 2022[36]	Ukraine	VR, heart rate variability, and anxiety	VR environment consists of a beach scene with some objects to view, such as sea waves, and a cat walking around	Bobo-VR X1 Headset	RCT	Healthy volunteers (N = 94; Group A: n = 56; Group B: n = 38 Group A: Age: Mean = 40.3 (SD = 9.2); Group B: Mean = 37.4 (SD = 9.6); Age Range: 20-60)	STAI ECG recordings of HRV	The headset was more effective in decreasing HF (p	Purr VR is a safe relaxation option for healthy individuals dealing with moderate stress.
Zhang et al. 2022[37]	China	VR and symptom distress (in breast cancer patients)	VR technology in the delivery of the CALM intervention	Head-mounted glasses and two controllers	RCT	Breast cancer patients receiving at least two courses of regular chemotherapy (N = 98 Age Range: 18-70)	FACT-B DT SAS SDS Concerns about Recurrences Scale (SCARS) PFS Pittsburgh Sleep Quality Index (PSQI)	VR-CALM group had significant changes in assessment scores before and after the intervention (quality of life (t = -10.38, p < 0.001), anxiety (t = 4.68, p < 0.001), distress (t = 4.10, p <0.001), concerns about recurrence (t = 2.74, p < 0.001) and fatigue (t = 9.913, p < 0.001)), whereas the Care As Usual group showed variations in some scores before and after CAU (quality of life (t = -2.99, p < 0.01), anxiety (t = 22124.82, p <0.001), concerns about recurrence (t = 2.37, p <0.05)).	VR-CALM was more effective than CAU, significantly reducing psychological distress and improving the quality of life for breast cancer survivors. Even in the 8-month follow-up, VR-CALM participants reported less distress, anxiety, depression, and sleep disorders. However, neither VR-CALM nor CAU significantly alleviated patients' fear of cancer recurrence.
Espinoza et al. 2012[38]	Spain	VR and induction of positive emotions in hospitalized oncology patients	VR to induce positive emotion	TV screen connected to a computer; keyboard and mouse; headphones	Pilot trial	Adult cancer patients with a Karnofsky functional state >/= 50, indicators of adequate organ function, life expectancy >/= 2 months and who were hospitalized for at least 1 week (N= 21 range= 41- 85, mean= 62.1, SD= 10.77)	HADS, Fordyce questionnaire VAS	Significant reductions in anxiety and depression (depression scale: t = 2.75; p=0.012; total HADS: t = 2.44, p=0.024) levels, along with increased happiness (happiness intensity: t = -2.12, p=.047; total happiness: t = -2.05, p<0.05) levels, were observed. Emotional state and physical discomfort improved after each VR session, with positive emotions increasing and negative emotions decreasing consistently across sessions, leading to enhanced mood, joy, and reduced anxiety and physical discomfort.	VR is a positive technology that can be used to promote well-being during hospitalization.
Schneider et al. 2003[39]	USA	VR and symptom distress (chemotherapy-related)	VR distraction intervention	Head-mounted device, which projects an image with the corresponding sounds; computer mouse for manipulating the image; commercially available headset	Pilot trial	Group of older women, aged 50-77, who were scheduled to receive intravenous chemo as part of their treatment plan (N = 16 Age: Mean = 57.7 (SD = 6.8) Age Range: 50-77)	PFS State Anxiety Inventory (SAI) SDS Open-ended Questionnaire	A significant SAI decrease (p = 0.10, Cohen's d = 0.44) occurred immediately after chemotherapy with VR. No significant changes in SDS or PFS (p>0.05). Although no significant changes were observed in symptoms of distress, fatigue, or anxiety 48 hours later, there was a trend toward lower scores with VR.	It is feasible to use VR distraction intervention in a population of older women. The intervention reduced treatment-related anxiety in this sample.
Schneider and Hood 2007[40]	USA	VR and symptom distress (chemotherapy-related)	VR distraction intervention	Commercially available headset (i-Glasses SVGA Head-Mounted Display, i-O Display Systems)	Pilot trial	Subjects were first diagnosed with breast, colon, or lung cancer and had a planned treatment, including at least two matched cycles of IV chemotherapy (N = 105 Mean age 54)	PQ ASDS-2 SAI PFS	VR didn't significantly impact symptoms of distress post-chemotherapy. While ASDS-2 and PFS declined more in the treatment group, differences weren't significant. SAI main effects were non-significant, but a significant crossover effect was seen (p=0.03). Higher VR interaction correlated (p<0.01) with lower symptom distress, shown by PQ and PFS (-0.296) and SAI (-0.308) correlations.	VR was a feasible distraction intervention in clinical settings, but it didn't reduce symptoms of distress during chemotherapy. However, a significant crossover effect was observed, indicating VR's effectiveness during initial, more anxious treatments, suggesting clinicians shouldn't assume VR will consistently improve chemotherapy-related symptoms.
Chirico et al. 2020[41]	Italy	VR and chemotherapy-related psychological distress	VR (explored an island), music therapy (relaxing music)	Head-mounted glasses with a head motion tracking system; controllers	RCT	Female patients with breast cancer who were having to receive chemotherapy with the same type of drug (N VR= 28, N music therapy= 30, N control=34 mean VR= 55.18, SD=5.7, mean MT= 55.7, SD= 5.26, mean control= 56.2, SD= 6.79)	SAI short version of Profile of Mood States (SV‐POMS) Virtual Reality Symptom Questionnaire (VRSQ)	VR and music therapy reduced anxiety levels significantly (p<0.001). Both interventions lowered negative mood states, including tension, depression, anger, and fatigue, while the control group didn't change significantly. Significant differences were seen between VR and control, and between music therapy and control.	Both VR and music therapy are useful interventions for alleviating anxiety and for improving mood states in breast cancer patients during chemotherapy. Moreover, the VR intervention seems more effective than MT in relieving anxiety, depression, and fatigue.
Fabi et al. 2022[42]	Italy	VR and psychological distress from chemotherapy	VR (relaxing nature videos)	3 VR headsets containing a selection of audiovisual productions made with 360-degree technology (Oculus Go); high-quality audio headphones	RCT	Patients with histological diagnosis of breast or ovarian cancer stage 1-3 (N = 44; VRE: n = 22; Control: n = 22 VRE Arm: 1) Breast Cancer: Age: Mean (Range) = 51 (37-71) 2) Gynaecological Cancer: Mean (Range) = 50 (36-61) Control Arm: 1) Breast Cancer: Mean (Range) = 50 (39-69) 2) Gynaecological Cancer: Mean (Range) = 52 (51-62))	HADs STAI Health-related Quality of Life (HRQoL)	HADs: VRE arm had stable distress scores at T1 and T3, with more in borderline and fewer in pathological ranges. STAI: At T1, the VRE group had higher state anxiety than controls. VRE group also showed a significant difference between STAI mean values (p>0.05), unlike the control group. Trait anxiety didn't differ significantly between groups (p>0.05). Quality of Life: No significant differences (p>0.05).	HAD results show no prevalent anxiety or depression disturbances in both groups, with mostly normal depression scores and similar trends over time. VRE had a limited impact on depression. For anxiety, VRE reduced pathological scores at T3 and borderline scores at T1 and T3, while the control group had increased borderline and pathological scores at T3. T5 analysis confirmed an improved psychological state in the VRE group.
Gullo et al. 2023[43]	Switzerland	VR and intervention/procedural distress	VR using a head-mounted display	The self-hypnosis device used was the OnComfort-Sedakit; it comprises an HMD mask Samsung Gear VR powered by Oculus with a Samsung S7 mobile phone or Pico G2, and headphones for tone and noise reduction	RCT	Participants were patients who were referred to the radiology department for peripheral endovascular interventions under local anaesthesia (N = 100 TAU: n=50; VA-HYPO: n=50 Age: Mean = 47.4 (SD = 16.8) Range: 18-84)	STAI VAS	Anxiety significantly decreased after the procedure, with VR showing a greater reduction (11.2 vs. 7 points, p<0.001) and a higher percentage of responders (76% vs. 46%, p=.004) compared to traditional treatment.	By significantly reducing pre-procedural anxiety, VR self-hypnosis has the potential to improve the management of a patient's distress. It is safe and effective for reducing anxiety during endovascular interventions.
Yang et al. 2019[44]	Korea	VR and anxiety reduction in patients undergoing arthroscopic knee surgery	VR (3-dimensional model of their own MRIs through a VR headset)	VR headset (Vive)	RCT	Patients expected to undergo elective arthroscopic knee surgery under general anaesthesia (N= 48, N non-VR= 24, N VR= 24 VR group: Age: Mean= 32. 5, range: 15-62, Non-VR group: Mean= 38, range: 20-65)	Amsterdam preoperative anxiety and information scale (APAIS) VAS	APAIS scores favoured the VR group, with significantly better outcomes in surgery-related anxiety (S) (p=0.014) and combined anxiety component (p<0.05) subscales. Preoperative VAS showed significantly higher satisfaction in the VR group, while postoperative VAS indicated better satisfaction and reduced stress for the VR group, with no significant differences in pain.	Preoperative VR experience reduces anxiety around surgical encounters.
Ganry et al. 2018[45]	France	VR and stress reduction in surgery patients	VR presenting natural scenes	Oculus VR glasses, audio headset	Pilot trial	Patients who were candidates for ambulatory skis cancer surgery in a university hospital (N=20 Age: mean= 56.9)	APAIS VAS salivary cortisol, HC	Significant decrease in VAS scores (anxiety) after VR treatment (p=0.009), significant decrease of salivary cortisol concentration after the VR test, not significant difference between pre and post-test HC.	The VR system seems to reduce preoperative anxiety, particularly with respect to the psychological and biological evaluation factors.
Brown et al. 2020[46]	USA	VR and peri-procedural pain and anxiety	Audiovisual monitor-flat screen, VR (relaxation video)	Oculus Go Headset	RCT	Patients electing to receive a lumbar spinal injection and patients meeting the definition of chronic LBP (N control= 13, N AV= 13, N VR = 14 Age: mean= 61.9, SD= 17.7)	PROMIS Modified Oswestry Disability Index (MODI) numeric pain rating (NPR) anxiety thermometer	No significant differences in baseline/post-injection pain or anxiety change scores among the three groups (p>0.05). Anxiety decreased least in control, followed by VR, with the greatest decrease in AV. A significant difference in baseline/pre-injection anxiety change between the control and audiovisual groups (p<0.05).	The AV and VR groups showed greater reductions in anxiety compared to the control group after the injection, but these differences weren't statistically significant. High within-subject variability made it challenging to establish statistical significance.
Almedhesh et al. 2022[47]	Saudi Arabia	VR and stress during caesarean section	VR (3D natural videos associated with calm Quran or music voices)	-	RCT	Low-risk pregnant women undergoing elective C-section with regional anaesthesia (N= 351, N control= 175, N intervention= 176 mean intervention= 31.20, mean control= 32.28)	HR, SBP, DBP, SpO2 B-MEPS NVFAS BSS-R	Significant differences were found between the VR and control groups in B-MEPS and NVFAS scores (B-MEPS: F(1) = 173.58, mean differences = -3.109, p < 0.001, 95% CI for d = (-3.57, -2.65), NVFAS: F(1) = 330.42, p < 0.001, mean differences = -2.11, 95% CI for d = (-2.35, -1.89)), with significant effects for time and time-group interaction. The VR group showed notably lower stress levels after skin suturing and two hours post-operation.	VR can reduce anxiety in women undergoing C-sections under regional anaesthesia. VR technique may generate positive feelings and mood improvements to decrease the patient’s anxiety before, during and after invasive procedures.
Vieira et al. 2018[48]	Portugal	VR and cardiac rehabilitation programme	VR, conventional environment	Kinect, booklet	RCT	Subjects who had just completed the phase 2 of Cardiac rehabilitation (N = 33). Intervention Group 1 (n = 11); Intervention Group 2 (n = 11); Control Group (n = 11) Age: Range: 40-75 IG1: Mean = 55 (SD = 9.0) IG2: Mean = 59 (SD = 11.3) Control Group (CG): Mean = 59 (SD = 5.8)	MoCA Trail Making Test (TMT) Verbal Digit Span (VDS) Stroop Test MacNew Questionnaire DASS 21	IG1 demonstrated significant Stroop Test score improvements, with significant differences between IG1 and IG2 in the M1-M2 variable (p<0.05). IG1 showed a significant increase in quality of life at M2, while IG2 exhibited emotional and social improvements at M1. No significant changes were observed in DASS 21 scores within groups (p>0.05).	When compared with the CG and the conventional format, the VR group presented improvements in executive function, specifically in selective attention and conflict resolution ability.
Rutkowski et al. 2021[49]	Poland	VR and pulmonary rehabilitation	VR (A head-mounted display)	VR TierOne device was used as the VR source; head-mounted display	RCT	For patients with a diagnosis of chronic obstructive pulmonary disease, pulmonary rehabilitation conducted in a ward setting (N = 50; VR Group: n = 25; CG: n = 25 VR Group: Mean = 64.4 (SD = 5.7); CG: Mean = 67.6 (SD = 9.4); Age Range: 45-85)	Perception of Stress questionnaire (PSQ) HADS 6-min Walk Test (6MWT)	The experimental group showed significant improvements in emotional tension, external stress (P<0.003), and total stress scores compared to the CG. They also exhibited significant improvements in HADS-A (p < 0.0009), HADS-D (p < 0.0001), and general HADS scores (p < 0.0001). Both groups saw significant improvements in exercise capacity, and the CG showed improved FEV1.	The VR group experienced significant reductions in stress levels, including emotional tension and external stress, with intermediate effect sizes. Additionally, they achieved statistically significant reductions in symptoms of anxiety, depression, and general psychological distress.
Jóźwik et al. 2022[50]	Poland	VR in reducing stress and depression and anxiety symptoms	VR-assisted therapy (VRT) (virtual therapy garden)	Oculus Rift S PC-Powered VR Headset	RCT	Low-risk pregnant women undergoing elective C-section with regional anaesthesia (N =34, N control= 11, N intervention= 23 Age: mean= 63.82, SD= 8.13)	HADS PSQ	The VR group had significant reductions in HADS total score (p=0.04), HADS-A (p=0.02), general stress score (p=0.01), emotional tension (p= 0.002), and external stress (p=0.03). Meanwhile, the CG experienced significant deterioration in overall stress score and intrapsychic stress.	VR has great potential to support cardiac rehabilitation by reducing the level of perceived stress and anxiety-depressive symptoms.
Lewandowski et al. 2021[51]	Poland	VR and symptom distress (Inflammatory bowel disease patients)	VR (played the gut healing application)	VR headset; controller	RCT	Patients with a confirmed diagnosis of inflammatory bowel disease and who remained on vedolizumab therapy (N = 90; Control: n = 45; Intervention group: n = 45 Mean Age (whole sample) = 34.7 (Sd= 9.9); Mean (Control) = 34 (SD = 9.8); Mean (Intervention) = 35.4 (SD = 10.9); Age Range: 20-60)	Questionnaire for assessing patient attitude to drug administration sessions, how user-friendly application is, subjective psychological indicators, sense of presence, symptoms related to use of simulator.	The intervention group exhibited significant improvements in well-being, relaxation (p = 0.046), treatment influence perception, drug efficacy perception (p < 0.001), positive attitude during treatment (p = 0.026), and motivation for treatment. However, no statistical significance was found for questions related to prolonged infusion time, calmness, and patient's influence during treatment.	Findings suggest a positive effect of VR during vedolizumab infusion. The implementation of VR intervention during vedolizumab infusion significantly increased understanding of the therapy process and reduced stress in the treated group.
Anderson et al. 2017[52]	USA	VR and relaxation	VR-presented natural settings (viewing three 15-minute 360 scenes, Control (i.e., empty indoor classrooms), Ireland, and Dream Beach)	Head-mounted display: Oculus Rift DK2, Oculus VR	Experimental study	General population (N = 18 Age: Mean = 32 (SD = 12))	Value of virtual reality questionnaire (VVR) Modified Reality Judgement and Presence Questionnaire (MRJPQ)	With time, the control scene significantly decreased positive affect (p=0.004), while Ireland and Dream Beach did not (p = 0.12 and p = 0.21 respectively). Negative effects significantly declined over time when viewing Ireland (p=0.005) and Dream Beach (p=0.03), but not the control scene (p=0.07). The PANAS results indicated a significant decrease in PA over time for the control scene but not for Ireland or Dream Beach. In the MRPJQ survey, participants' agreement increased significantly for statements about the benefits of using the VR system and its ability to improve relaxation after the experiment.	Natural scenes delivered via VR provide relaxation and restoration after a stressful experience. Subjects found the VR experience to be positive, which was reflected in their change in attitude toward VR.
Zolfaghari et al., 2022[53]	Australia	VR and stress in emergency departments	VR intervention for between 20 and 40 min	Netflix TV Shows with a G or PG rating (e.g. Big Bang Theory, Glee), a mindfulness app (TRIPP) and a non‐violent game (Tetris)	RCT	Patients who were ambulatory (N=32 Mean age: 16.8, SD: 1.91)	STAI‐S‐F SSSQ‐D	A significant reduction in distress as measured by the SSSQ‐D, from 13.1 (SD 5.5) pre‐intervention to 9.4 (SD 3.1) post‐intervention (t = 4.55, P	VR technology can effectively be used in EDs to assist adolescents and young adults in better managing their distress.
Shah et al., 2015[54]	USA	VR and stress in people with mood disorders	VR and psychoeducation	Three daily 1-hour sessions incorporating psychoeducation and VR-based relaxation practice	Single-group, pretest–post-test, quasi-experimental research design	People with mood disorders (N=22, aged between 21 and 65 years)	SUD	Participants who completed the programme had significantly lowered subjective stress (t = 6.91, p < 0.001), depression (t = 5.62, p < 0.001), and anxiety (t = 5.54, p < 0.001); and increased skin temperature (F = 17.71, p < 0.001), perceived relaxation (F = 26.20, p < 0.001).	VR technology is beneficial for individuals with mood disorders.
Wang et al., 2020[55]	Switzerland	VR and generalized anxiety disorder	Virtual nature or a virtual abstract painting	The VE system simulated outdoor cycling in an immersive environment. It projected content around participants using 3D projection technology on multiple screens.	RCT	Patients with generalized anxiety disorder (N=77 VN Group (n = 40) Mean age: 58.43 (SD 7.37) VAP Group (n = 37) Mean age: 59.87 (SD 6.99))	PSS	The significant main effect of Time (F(1,75) = 30.97, p < 0.001, ηp2 = 0.30)) and Group (F(1, 75) = 6.63, p < 0.001, ηp2 = 0.15), indicating that the post-exercise perceived stress scores (4.1 ± 0.07) were higher than the pre-exercise values (3.40 ± 0.10) across the two groups, and the perceived stress scores for the VN group (3.88 ± 0.09) were higher than those for the VAP group (3.58 ± 0.09) across the two time points.	A virtual exercise environment is an effective way to induce a relaxing effect in patients with generalized anxiety disorder.
Afifi et al., 2023[56]	England	VR and older adults with mild cognitive impairment	3 sessions of VR	In VR Session 1, participants chose five travel adventures from 25 options. Session 2 revisited past locations using Google Street View, with addresses provided beforehand. In Session 3 approximately 15 family photos and 1 video were uploaded and viewed together on a virtual couch.	Clinical trial	Senior living community with dementia (N=21 Mean age: 83.10, SD = 9.76)	PSS	The VR was associated with improvements in older adults’ affect and stress (p=0.43), relationship with their family members, and overall quality of life, compared to baseline.	Older adults with dementia and their family members might benefit even more from using the VR than older adults with MCI and their family members.
Szczepanska-Gieracha et al., 2021[57]	USA	VR and stress in patients undergoing cardiac rehabilitation	Eight 20-minute VR therapy sessions, conducted twice weekly for four weeks	The VRTierOne® device by Stolgraf, employing VR HTC VIVE goggles and controllers, was used for therapy. It provided immersive stimulation through visual, auditory, and kinesthetic experiences. The Virtual Therapeutic Garden, based on Ericksonian psychotherapy, featured symbols like the Garden of Revival, representing the patient's health journey.	RCT	Patients with Coronary artery disease (N=34 (mean age: 68.91, SD=6.26, N=17 in each group))	PSQ HADS	In the intervention group, a significant decrease in HADS score was observed (19.46 pretreatment vs. 15.73 post-treatment, p = 0.003), HADS-Anxiety subscale decreased by 16.0% (p = 0.01) and HADS-Depression by 23.0% (p = 0.003). Similarly, a significant decrease in PSQ was recorded at 12.8% (64.73 vs. 56.47, p = 0.03).	Immersive VR therapy effectively supports the CR of individuals with anxiety-depressive symptoms.
Veling et al., 2021[58]	Canada	VR and stress in patients with a psychiatric disorder	VRelax or standard relaxation	VRelax used a Samsung Galaxy S6 or S7 smartphone connected to a Samsung Gear VR headset. Three-dimensional audio played through headphones. Participants began in a waiting room for presession measures before transitioning to 360-degree nature videos, navigated by looking at hotspots. Scenes included beaches, coral reefs, underwater dolphin encounters, drone flights, mountain meadows, Alpine scenery, cliffside views, and Tibetan singing bowl therapy sessions.	RCT	N=50 patients receiving ambulatory treatment for anxiety, psychotic, depressive, or bipolar disorder Mean age: 41.6 (SD=14.2)	PSS VAS	Symptoms of depression and anxiety were reduced significantly after VRelax use. Perceived global stress levels, symptoms of depression, and paranoid thoughts were significantly lower after standard relaxation exercises.	VRelax may provide a much-needed, effective, easy-to-use self-management relaxation intervention to enhance psychiatric treatments.
Blum et al. 2019[59]	Germany	VR and stress	VR-based heart rate variability biofeedback	Virtual beach scenery at sunset	RCT	Healthy volunteers (N=60 Mean age: 33.5 SD = 9.4)	STAI Stroop tasks	Stroop tasks reduced and both treatments increased relaxation. There was no between-groups difference in relaxation during the treatment.	VR-based implementation buffered perceived stress in the subsequent stressor task, increased relaxation self-efficacy more, reduced mind wandering, helped participants focus on the present moment, and helped preserve attentional resources.
Pallavicini et al. 2009[60]	Italy	VR and GAD	Biofeedback-enhanced VR system and mobile phone	In the virtual reality + mobile phone without biofeedback condition (VRM), patients underwent an eight-session VR-based treatment involving relaxation and exposure techniques. They experienced serene environments like Campfire, Beach, and Waterfall, using a mobile phone at home to practice relaxation daily. In the virtual reality + mobile phone with biofeedback condition (VRMB), patients had the same protocol with heart rate variations modifying aspects of the virtual environment during therapy sessions.	RCT	GAD patients Seeking treatment in a public healthcare institute (N=12, age between 18 and 50)	STAI HAM-A GAD-7	Subjects belonging to this group reported a higher decrease in some of the anxiety psychometric questionnaires after the treatment than both VRM and WL groups, even if the VRM group, too, reported some significant improvements at the end of therapy.	VR can also be used in the treatment of GAD, secondly, in a VR treatment, patients can take advantage of a mobile device that delivers guided experiences similar to those experienced in VR in an outpatient setting.
Venuturupalli et al. 2019[61]	USA	VR and chronic pain and anxiety of rheumatology outpatients	Guided meditation environment and a respiratory biofeedback	Samsung Gear VR goggles; Samsung Galaxy S7; Nubwo N2 headphones	RCT	Adult patients with physician-diagnosed chronic autoimmune disorders who were on a stable medication regimen (N=17 Age: mean= 52.65, SD= 16.1)	PROMIS Emotional Distress/Anxiety Emotional Distress/Anger Global Health Scale, VAS FAS	Pain was significantly reduced after both BFD (mean reduction = 1.07, t = 2.83, d = 0.50, p = 0.01) and GM (mean reduction = 1.09, t = 2.29, d = 0.52, p = 0.04). Significant differences were observed in FAS scores at baseline, after BFD, and after GM. The most substantial anxiety score reduction occurred after the GM intervention (mean reduction = 0.59, t = 2.28, d = 0.91, p <0.05).	VR could be a feasible nonpharmacological solution for the management of pain and anxiety in rheumatology patients.
Meyer et al. 2022[62]	Germany	VR and psychological distress from Covid-19	VR video Secret Garden + a series of social exercises	Simple cardboard VR headsets (Basetech Headmount Google 3D), compatible with smartphone displays were utilized; head-mounted display	Pilot trial	General population (N = 38 Age: Mean = 36.4 (SD = 12.5) Range: 20-67)	PSS-10 DASS SCS BHS Fear of COVID-19 Scale (FCV-19S) SRSI3 SUDs	Significant changes over four measurements (day -7, 0, 7, and 21) were observed in general distress, depression, anxiety, stress, and perceived stress. Hopelessness remained unchanged. Improvement occurred from day 0 to 7 (p < 0.01) and on day 21, general distress and these subscales were significantly lower than on day 0. Participants improved in social connectedness from day 0 to 7 (p<0.01). Subjective distress differed significantly between days 1 and 7 for state measures.	VR-based self-help interventions can help mitigate the psychological burden associated with the pandemic. These self-guided interventions can help isolated individuals manage depression, stress, anxiety, negative feelings and well-being at home during the COVID-19 pandemic.
Farahimanesh et al. 2023 [63]	Iran	VR and psychological distress from COVID-19	VR video Secret Garden + series of social exercises	A software called Unreal Engine was used to create the VR environment; head-mounted display/headset	RCT	General population COVID group: N = 30 CG: N = 30 COVID Group: Age: Mean = 49.1 (SD = 10.92) Control: Mean = 49.70 (SD = 10.40)	DASS-21 PSS BHS SCS FCOR	COVID group showed significant reductions in perceived stress, depressive symptoms (p<0.05), and anxiety levels at T1, maintained at follow-up, while the CG exhibited no changes (p>0.05). There were no significant differences in hopelessness or fear of COVID-19 between groups or across time points (p>0.05).	The VR self-help COVID group was feasible and effective at reducing the psychological distress experienced during the COVID-19 pandemic. The gains obtained in the COVID group were maintained throughout the 2 weeks of follow-up.
Riva et al. 2021[64]	Italy	VR and COVID-19 anxiety	VR video Secret Garden + series of social exercises	Head-mounted display or low-cost cardboard VR headset	Pilot trial	Non-smoking, healthy men (N=40 Age: mean= 30.28, SD=11.69)	DASS-21 PSS BHS SCS FCOR STAI SRSI3 SUDs	Participants improved in depression, stress, distress, and perceived stress from T0 to T1, maintained at T2 (p<0.05). Anxiety, refreshment, energy, relaxation, peace, quiet, somatic, emotional, cognitive distress and discomfort showed time effects.	A potential benefit of the intervention (COVID-19 Feel Good) in reducing psychological distress in participants who had experienced at least two months of strict social distancing measures.
Vlake et al. 2022[65]	Netherlands	VR and COVID-19	14-minute-long video that was watched using VR, featuring the ICU environment along with voice-over explanations	Head-mounted display--VR glasses (Oculus Go) and headphones	RCT	Adult patients who were treated in an ICU of one of the participating hospitals and visited the COVID-19 post-ICU follow-up clinic (N = 89; ICU-VR: n = 45; Control: n = 44 Age: Mean = 58 (SD = 11); ICU-VR: Mean (Median (IQR)) = 61(54-65); Control: Mean = 59(51-65))	IES-R HADS EQ-5D Questionnaire to assess quality and satisfaction with ICU aftercare	Psychological distress and quality of life remained limited throughout the follow-up, with no differences between the two groups. However, ICU-VR patients expressed higher satisfaction with ICU aftercare and rated it more favourably compared to controls. Anxiety significantly reduced in the VR group at first (OR 3.3, 95% CI 1.2 - 9.3, p = 0.02) and second (18% vs 50%; OR 3.8, 95% CI 1.1 - 12.7; p = 0.03) timepoints.	ICU-VR improved the perceived quality of life and satisfaction with ICU aftercare among COVID-19 ICU survivors. While it was feasible and acceptable, approximately 31% of COVID-19 ICU survivors experienced ongoing psychological distress up to 6 months after hospital discharge, and ICU-VR did not significantly enhance psychological recovery or quality of life.
Beverly et al. 2022[66]	USA	VR and stress reduction in healthcare workers in COVID-19 treatment units	360-degree video capture of a lush, green nature preserve	Statulator (an online statistical calculator) to conduct a priori power analysis. Cine-VR simulations were screened in an Oculus Go or Pico G2 4K HMD.	Pilot trial	Frontline healthcare workers, including direct care providers, indirect care providers, and support or administrative services (N= 101 Age range 25-34)	VAS	Significant reduction in subjective stress from pre to post-simulation (mean change = -2.2±1.7, t = 12.749, p < 0.001, Cohen’s d of 1.08), those who met the cut-off for high-stress pre-simulation showed a greater reduction in subjective stress scores.	VR simulation had a meaningful impact on subjective stress. Findings support the use of the Tranquil Cine-VR in reducing subjective stress in the short term among frontline healthcare workers.
Croghan et al. 2022[67]	USA	VR and psychological distress from COVID-19	VR (two videos, “walk in the wood” and “forest of focus”, were displayed through 3-C VR and with computer 4K graphic imagery)	Reulay Oculus headgear	Pilot trial	Healthcare providers in the frontline of the COVID-19 pandemic (N = 24 Age: Mean = 46 (SD = 10.5))	STAI-Y1 to Brief Resilience Scale (BRS6) PROMIS Emotional Distress-short form PROMIS Cognitive Function short form Self-efficacy WIWI Reulay Qualitative Survey (RQS)	VR walk in the woods had the greatest pre-to-post change (6.4 points), followed by VR forest of focus (5.8 points), Computer Screen Forest of focus (5 points), and Computer Screen Walk in the Woods (4.1 points). All sessions showed significant score decreases (p<0.005). Emotional distress and cognitive function scores improved in all sessions.	This pilot study of nature-based guided imagery experiences has shown that 10 minutes of VR experience has the potential to reduce anxiety and emotional distress as well as enhancing focus on healthcare providers.
Ho et al. 2023[68]	Taiwan	VR on factory employees' stress	30-minute VR nature-based videos	VR headset (Oculus Quest 2, META, US)	Clinical trial	Factory workers who had break time in the afternoon (N=35, N intervention= 18, N control= 17 Age: mean VR= 55.21, SD VR= 7.71_ mean control= 36.10, SD control= 11.12)	Four-Dimensional Symptom Questionnaire (4DSQ) PANAS PSS SBP DBP HRV ANS	Significant group effects and group-by-time interactions were observed for distress (p=0.021) and anxiety (p=0.039) in psychological measures. Mean differences indicated improvements in distress, depression, anxiety, and positive affect in the VR group following the intervention compared to the comparison group. However, somatization, negative affect, and perceived stress did not show significant effects (p>0.05).	VR natural experiences had positive effects on psychological stress of factory workers, including distress, depression, anxiety, and positive affect, but did not have an effect on perceived stress.
Weitzman et al. 2021[69]	USA	VR, stress management and burnout in Otolaryngology residents	10-minute sessions of weekly VR-guided meditation (i.e., paced breathing and mindfulness exercises)	Software combined a smartphone (Galaxy S7, Samsung Inc., South Korea) fitted to a Samsung Gear Oculus VR headset	RCT	Otolaryngology Residents (N=18 Range: 17 between 26 and 30 and 1 between 31 and 35)	Maslach Burnout Index with three subscales: emotional exhaustion, depersonalization, and personal accomplishment	Weekly use of VR-guided meditation and paced breathing was associated with a significant decrease in the EE subscale score (p=0.009). VR was not associated with significant changes in the DP or PA subscale scores.	VR-based mindfulness meditation: this tool was a welcomed addition among residents that successfully decreased burnout in Otolaryngology residents.
O’Gara et al., 2022[70]	England	VR and compassionate mind training to support oncology patients	VR intervention accompanied by Compassionate Mind Training exercises over a period of six months	Head-mounted, stand-alone VR device	Two-phase study design	Adults diagnosed with cancer and undergoing treatment, recovery, or palliative care (N = 20 Range: 22-77 Mean = 48.7 SD = 16.87)	EQ-5D/QLQ-C30, Profile of Mood Scale, Warwick and Edinburgh Mental Health Well-being Scale, DASS-21, SCS, AAQ, Locally Developed Questionnaire	Mental well-being improved following each use and from baseline to the conclusion of Session 3 (VR 1-z = 2.846, p≤0.01; VR 2-z = 2.501, p≤0.01; VR 3-z = 2.492, p≤0.01). A statistically significant difference in the mean scores was demonstrated for EDA at mid-session and post-session in comparison to the presession (F (1.658, 4.973) = 13.364, p≤0.05)	SafeSpace was concluded to be an acceptable and feasible intervention with positive effects on mental well-being/stress in the oncology setting.
Vaquero-Blasco et al., 2021[71]	Switzerland	360-degree VR experiences as a tool for stress relief	VR exposure for a total of ~18 minutes. RS1, followed by the MIST, followed by RELAX, and concluding with RS2.	Oculus Quest HMD and EEG	RCT	Healthy adult participants were included in this study. (N = 23 Range: 18-40 Mean = 22.65)	EEG SPSL	The results of this study validate the effectiveness of 360-degree VR experiences in significantly reducing stress (p = 0.0001). A positive correlation (r = 0.8417) was found between the SPSL answers and the average RG. Participants reported high levels of satisfaction with the VR experiences and expressed a willingness to repeat the intervention.	VR-based 360-degree experiences were effective in reducing stress levels among healthy participants. This study suggests that 360-degree VR experiences can act as a stress-relieving tool for the general public.
Chiu et al., 2023[72]	Hong Kong	VR and reduce preoperative anxiety	360-degree VR video/virtual tour depicting the preoperative process	Oculus Quest 2 HMD	RCT	Adults who were scheduled for their first elective surgery procedure under general anaesthesia. (N = 74, N control = 37, N intervention = 37 Range: 18-69 Mean = 46.34 SD = 14.52)	APAIS VAS Simulation Sickness Questionnaire	VR-based intervention group displayed significantly decreased preoperative anxiety at T1 (β, -5.46; 95% CI, -7.60 to -3.32; p＜.001) and T2 (β, - 5.57; 95% CI, -7.73 to -3.41; p＜.001), lower stress at T1 (β, -10.68; 95% CI, -16.00 to -5.36; p＜.001) and T2 (β, - 5.16; 95% CI, -9.87 to -0.45; p =.03). Satisfaction levels were significantly increased in the intervention group in comparison to the CG (mean [SD] score, 81.35 (9.24) vs 65.28 (8.16); difference, 16.07; 95% CI, 12.00 to 20.15; p＜0.001).	The intervention was found to be effective in reducing anxiety levels and stress while improving preparedness in adults scheduled for their first elective surgery. While there was no significant impact on pain levels or length of stay, the results support the feasibility and effectiveness of using VR-based technology for patient education and surgery preparation.
Cheng et al., 2020[73]	Canada	VR and stress in older adults	3D VR and hands-on aromatherapy	The intervention comprised 2-hour weekly sessions across 9 weeks. In the first week, ice-breaking activities introduced participants to wearing 3D VR helmets and operating VR handles with familiar scenes	Quasi-experimental	Institutionalized elderly participants (N=60 mean age: 85.2)	PSS	The experimental group showed significant post-intervention improvements in terms of scores for happiness, perceived stress, sleep quality, meditation experience, and life satisfaction (n=48; all P<0.001).	3D VR and hands-on aromatherapy promote psychological health in institutionalized older adults.
Gaggioli et al., 2014 [74]	Canada	VR and psychological stress	Virtual scenarios	Immersive scenarios, created with NeuroVR 2, involved role-playing and relaxation environments. Participants received free technology and biosensors for stress monitoring, along with training. Biofeedback in therapy sessions adjusted virtual experiences based on physiological data. Wireless sensors recorded real-time physiological data, analysed for stress tracking.	RCT	Teachers and nurses (N=60 aged 25-60 years)	STAI PSS	Stress scales (PSS) analysis revealed a significant reduction in both treatment groups. Significant differences in the STAI Y2 reduction (Teachers: -0.44+/-8.9; Nurses: -3.2+/-5.3; P=0.44, effect size=0.04)	Interreality protocol yields better outcomes than the traditionally accepted gold standard for psychological stress treatment.
Dings et al., 2021[75]	England	VR and stress during the vasectomy	Two-dimensional video glasses or VR glasses	Experimental Group 1 used 2D video glasses showing a nature movie about coastlines and the sea with relaxing music. The content was displayed on a 2D screen inside the glasses for consistency. Experimental Group 2 used Samsung Gear VR glasses, isolating them. They watched four short 360° movies: three nature scenes and a short film depicting a couple at home. The total content duration was 28 minutes.	RCT	Patients planned for a vasectomy (N=140 N=61 CG (mean age=43), N=43 in the 2D video glasses group (mean age=40) N=36 VR group (mean age=41))	VAS STAI-AD	Patients in the VR group experienced significantly more anxiety during the procedure (OR 1.40, 95% CI 1.07–1.85). Also, patients without prior hospitalization reported significantly more pain than patients with one or more hospitalisations (OR 1.35, 95% CI1.11–1.65).	The VR and 2D video glasses did not reduce pain or stress during the vasectomy. In the VR group, the anxiety levels during the procedure were even higher.

Included Studies Characteristics

The studies included in the review exhibit a broad distribution of characteristics, encompassing diverse populations, interventions, methodologies, and outcomes. Across the spectrum of populations, participants ranged from older to younger adults, including clinical populations grappling with PTSD, anxiety disorders, cancer, cardiac diseases, autoimmune disorders, and other chronic conditions, as well as non-clinical populations such as teachers, nurses and healthy volunteers. Interventions varied widely, spanning VR exposure therapy (VRE), VR-based relaxation techniques such as guided meditation and respiratory biofeedback, exercise programmes for cardiac rehabilitation, distraction interventions during medical procedures, social support interventions, and VR self-training for specific disorders such as social anxiety disorder (SAD) and generalized anxiety disorder (GAD). Studies were characterized by diverse designs, including randomized controlled trials (RCTs), multi-centre trials, and secondary analyses of existing datasets. Treatment durations varied from single-session interventions to multi-session programmes. Technologically, VR interventions utilized headsets, immersive environments, and interactive applications tailored to specific intervention goals, often integrating biofeedback sensors to monitor and customize interventions based on individual needs. The quality of the included studies was generally good, with a low risk of bias (Appendices Tables 2-3).

Three pivotal studies delved into the efficacy of VR interventions in mitigating stress outcomes in individuals grappling with PTSD. Reger et al. scrutinized the comparative effectiveness of prolonged exposure therapy (PE) versus VRE therapy among United States army soldiers contending with PTSD stemming from deployment trauma. While both interventions showed promise in reducing stress symptoms, there were no discernible differences in their efficacy, underscoring the potential of VR as a viable therapeutic tool [26]. Nor et al. furthered this inquiry by conducting a secondary analysis on military personnel undergoing PE or VRE for combat-related PTSD. Their findings not only reaffirmed the significant stress symptom reduction across both treatment modalities but also shed light on the pivotal role of in-vivo exposure in driving therapeutic outcomes [27]. Additionally, Loucks et al. delved into the application of VRE therapy for veterans with PTSD resulting from military sexual trauma. Their study underscored the substantive reduction in stress symptoms post-treatment and sustained improvements at follow-up, accentuating the transformative potential of VR interventions in alleviating stress burdens among this vulnerable population [28]. Together, these investigations underscore the burgeoning promise of VR-based interventions in ameliorating stress outcomes in individuals grappling with PTSD, offering a beacon of hope in the landscape of trauma-focused therapy.

Several studies have examined the efficacy of VR interventions in managing stress across various populations. Kim et al. investigated the impact of VR self-training (VRS) on individuals diagnosed with SAD. Participants engaged in VR speech rehearsals, leading to significant decreases in self-reported anxiety, avoidance scores, distress, and negative self-evaluation [29]. Freeman et al. assessed distress reduction in participants with persecutory delusions during VRE therapy, finding gradual distress reduction across sessions, particularly in the group undergoing VR cognitive therapy [30]. Hoch et al. investigated stress-reduction techniques in an online virtual world. The authors designed a translated version of an existing 8-week relaxation response-based resilience programme in VR. The general trend was toward improvement using the PSS and the Symptom Checklist 90- Revised (SCL-90-R) [31].

Richesin et al. compared stress management interventions involving classic 2D art-making, novel 3-dimensional (3D) VR art-making, and a non-artistic VR CG, finding significant decreases in negative affect, state anxiety, trait anxiety, and perceived stress post-VR intervention [32]. Kothgassner et al. examined the impact of virtual social support on stress levels, reporting that avatar and real social support reduced stress and promoted prosocial behaviours compared to agent-based or no-support conditions [33]. Villani et al. evaluated stress management techniques using VR, video, and audio mediums, observing significant reductions in stress in the VR condition compared to video and audio, indicating lower physiological arousal [34].

Tan et al. investigated stress management among participants with mental disorders using a VR programme called V-DESSERTS, reporting lower subjective stress, higher perceived relaxation, and greater improvement in knowledge compared to the control group [35]. Aganov et al. explored the effectiveness of Pure Purr technology in reducing stress levels among healthy adults, finding enhanced parasympathetic activity and decreased sympathetic activity with the investigational device compared to the sham device. Moreover, State-Trait Anxiety Inventory (STAI) scores decreased after a 5-minute VRE to either headset [36]. Zhang et al. investigated VR-Managing Cancer And Living Meaningfully (CALM) intervention's impact on stress outcomes among breast cancer patients undergoing chemotherapy, finding significant reductions in distress levels, anxiety, and depression scores [37]. Espinoza et al. aimed to promote emotional well-being using VR interventions among adult cancer patients, reporting significant reductions in anxiety and depression levels post-intervention, along with increased happiness levels [38]. Schneider et al. explored the efficacy of VR distraction intervention in alleviating chemotherapy-related symptom distress among older women with breast cancer, finding a significant reduction in anxiety scores immediately after treatment with VR [39]. However, in the other study, Schneider et al did not find any significant differences in measures of symptom distress immediately following chemotherapy and or at two days follow-up [40]. Chirico et al. assessed the impact of VR and music therapy on anxiety levels in breast cancer patients undergoing chemotherapy, observing significant reductions in stress, anxiety and negative mood states post-intervention [41]. Fabi et al. investigated distraction therapy using VRE in breast or ovarian cancer patients undergoing chemotherapy, reporting significant reductions in state anxiety scores in the VRE group compared to controls immediately after and within 48 hours of chemotherapy [42]. These studies collectively underscore the promising role of VR interventions in alleviating stress and promoting emotional well-being across diverse populations, ranging from individuals with mental disorders to cancer patients undergoing chemotherapy and healthy adults.

Gullo et al. evaluated the effectiveness of VR-augmented self-hypnosis in reducing anxiety during peripheral endovascular interventions, finding significantly lower anxiety after the procedure in the virtually augmented self-hypnosis group compared to treatment as usual [43]. Yang et al. explored the impact of preoperative VRE to 3D reconstructed magnetic resonance images on anxiety reduction in patients undergoing arthroscopic knee surgery, reporting significantly lower surgery-related anxiety and higher satisfaction in the VR group compared to the non-VR group [44]. Ganry et al. observed a significant decrease in anxiety scores after immersive VR experience among individuals with anticipated high stress levels during surgery [45]. Brown et al. investigated the impact of VR interventions on stress outcomes in chronic low back pain patients undergoing spinal injections, finding potential benefits of audiovisual interventions in reducing anxiety during medical procedures [46]. Almedhesh et al. assessed the impact of VR on maternal anxiety and stress levels during elective caesarean section, reporting significantly reduced stress and anxiety levels in the VR intervention group compared to controls [47]. Vieira et al. examined the effect of a VR exercise programme on stress levels in patients undergoing cardiac rehabilitation, observing a significant reduction in stress levels compared to the control group [48]. Rutkowski et al. investigated the effectiveness of VR-led pulmonary rehabilitation in reducing stress levels in patients with Post-Acute Sequelae of SARS-CoV-2 infection, finding significant reductions in stress levels post-rehabilitation [49]. Jóźwik et al. reported significant improvements in stress levels among coronary heart disease patients undergoing VR therapy compared to standard care [50]. These findings collectively highlight the potential of VR interventions as effective tools for managing anxiety and reducing stress across various medical procedures and patient populations.

Lewandowski et al. explored the impact of VR immersion during vedolizumab infusion on stress outcomes in patients with inflammatory bowel disease, finding significant improvements in stress-related outcomes in the VR group compared to the control group [51]. Anderson et al. observed significant reductions in stress levels during exposure to natural VR scenes compared to control scenes, as indicated by outcome measures and subjective mood assessments [52]. Zolfaghari et al. reported notable reductions in distress and negative affect post-VR intervention among young patients in emergency departments [53]. Shah et al. found significant reductions in stress, depression, and anxiety levels post-VR intervention among inpatients with major depressive disorder and bipolar disorder [54]. These studies collectively suggest that VR interventions hold promise in effectively managing stress across various populations and settings. Wang et al. investigated the impact of virtual environments on stress relief in individuals with GAD [55]. Seventy-seven participants were randomly assigned to either a virtual nature (VN) or virtual abstract painting (VAP) group. Both groups engaged in 20-minute cycling sessions in VR environments. Post-exercise alpha values, indicating relaxation, were significantly higher in the VN group compared to the VAP group. While perceived stress scores were higher post-exercise, the VN group reported greater stress relief than the VAP group. Moreover, the VN group showed significantly higher levels of restorative quality and satisfaction after the intervention, suggesting that exposure to VR natural environments may offer superior stress relief and enhance psychological well-being in individuals with GAD compared to abstract paintings [55]. Afifi et al. conducted a study involving 21 family dyads from a senior living community to assess the effects of VR sessions on stress outcomes for older adults and their family members [56]. Participants engaged in VR sessions designed to evoke positive memories and experiences, while self-report measures were used to evaluate stress levels. Results indicated significant reductions in perceived stress for both older adults and family members following the VR sessions [56]. Additionally, family members reported decreased caregiver burden, highlighting the potential benefits of VR interventions in mitigating stress among older adults and their caregivers. These findings suggest that VR technology may offer a promising approach to addressing stress-related challenges in ageing populations and their families.

Szczepanska-Gieracha et al. examined the effects of VR therapy on stress outcomes in 34 patients undergoing cardiac rehabilitation (CR) for coronary artery disease (CAD) [57]. The study compared VR therapy with standard relaxation techniques. Results revealed significant reductions in anxiety, depression, and perceived stress among patients receiving VR therapy compared to the control group [57]. These findings suggest that VR therapy may effectively alleviate stress in CAD patients undergoing CR, potentially enhancing their overall psychological well-being and treatment outcomes.

In another study by Veling et al., psychiatric patients undergoing stress-reducing interventions, including the VRelax app and standard relaxation exercises, experienced significant reductions in negative affective states and improvements in positive affective states. VRelax demonstrated a larger beneficial effect on negative affective states compared to standard relaxation exercises, with short-term effects including reductions in depression and anxiety symptoms [58]. These findings underscore the potential of VR-based interventions like VRelax in alleviating stress outcomes across various psychiatric disorders. Blum et al. investigated the effects of standard heart rate variability biofeedback (Standard-BF) and VR-based heart rate variability biofeedback (VR-BF) treatments on relaxation in healthy participants [59]. Both groups received a single 10-minute session of heart rate variability biofeedback involving slow diaphragmatic breathing. The VR-BF group experienced a virtual beach scenery while practising biofeedback, while the Standard-BF group received abstract graphical feedback. Subjective relaxation was measured using the STAI. Results showed that both treatments increased relaxation, as indicated by reduced STAI scores after each stressor and a reduction in scores from before to after the treatment session [59]. Overall, the study partially confirmed the hypothesis that VR-BF would lead to greater relaxation compared to Standard-BF. Pallavicini et al. aimed to test the INTREPID project approach in a Phase II randomized controlled trial with GAD patients [60]. Treatment groups underwent eight sessions of VR-based treatment with or without biofeedback. Results showed significant improvements in stress outcomes across groups. Participants reported high satisfaction with the treatment [60]. Finally, Venuturupalli et al. conducted a study on 20 participants with chronic autoimmune disorders [61]. They underwent VR sessions with guided meditation (GM) and respiratory biofeedback (BFD). Both interventions significantly reduced pain, stress and anxiety levels. GM was more effective in reducing anxiety. The order of intervention did not significantly affect outcomes [61]. Overall, the study highlighted the potential of VR interventions in managing stress outcomes in patients with chronic autoimmune disorders.

Two RCTs and a pilot study [62,63] with a within-subject design [64] evaluated the efficacy of a VR-based self-help intervention “COVID Feel Good” in Germany, Iran, and Italy, respectively. They used various scales to measure outcomes such as stress, depression, and anxiety [62-64] In general, these interventions led to significant improvements in stress, depression, and perceived stress, but not in hopelessness [62-64]. Another study examined the feasibility of an ICU-specific VR intervention [65]. One month after the intervention, fewer participants in the VR group reported probable anxiety compared to the control group. However, anxiety scores were not significantly lower in the VR group at this point or at the three-month follow-up.

Regarding stress among healthcare workers during COVID-19, two studies explored VR interventions [66,67]. One study showed a significant reduction in stress among frontline healthcare workers after a short VR simulation of a nature scene [66]. The other study involved healthcare professionals viewing nature-based content. Concerning anxiety, the VR walk in the woods was the most effective in diminishing anxiety from pre- to post-intervention, followed by VR forest of focus, computer screen forest of focus, and computer screen walk in the woods. All groups experienced a decrease in anxiety, but no significant differences between the groups [67]. These studies underscore the potential of VR-based interventions in addressing mental health challenges during the COVID-19 pandemic and in ICU settings.

The study conducted by Ho et al. aimed to evaluate the effects of a VR intervention on stress-related outcomes among factory workers in Taiwan [68]. The VR intervention involved watching nature-based VR videos for 30 minutes once a week for 12 weeks during the workers' break time, while the comparison group received no interventions. The results showed significant improvements in psychological measures such as distress, anxiety, depression, and positive affect in the VR group compared to the comparison group [68]. These findings suggest that the VR intervention had beneficial effects on stress reduction among factory workers. The study conducted by Weitzman et al. focused on evaluating the effects of a VR-GM intervention on burnout among Otolaryngology residents [69]. Participants were randomized into two groups, with one group undergoing the VR intervention using a smartphone app for 10 minutes weekly, while the other group served as the control [69]. Results showed a significant decrease in the emotional exhaustion subscale score after the intervention, indicating reduced burnout. Subgroup analysis revealed that the male gender was associated with a decrease in EE subscale score, whereas the female gender was not. Overall, participants rated the VR-GM as enjoyable and easy to use, with potential usefulness as a stress management tool.

The study conducted by O'Gara et al. investigated the acceptability and feasibility of a novel intervention utilizing VR among cancer patients [70]. The findings revealed satisfactory acceptability of the intervention, with most participants completing all three sessions. Further analyses indicated improvements in mood and a reduction in stress levels following the intervention sessions. Overall, the study provided valuable insights into the feasibility and acceptability of the VR intervention among cancer patients, suggesting its potential utility in reducing stress levels and enhancing psychological well-being in this population [70]. The study by Vaquero-Blasco et al. examined the impact of a 360-degree VR relaxation experience on stress levels in healthy participants [71]. The session involved EEG recording and consisted of phases: initial resting state, a stress-inducing arithmetic test, 5-minute VR relaxation with scenarios such as beach and space, and a final resting state. Results indicated a significant reduction in self-reported stress levels post-VR relaxation, corroborated by EEG biomarkers such as relative gamma showing an overall decrease during the relaxation phase. However, individual responses varied, with some participants showing direct RG-stress relationships and others inverse [71].

Chiu et al. conducted a study to assess the impact of a VR intervention on preoperative anxiety among patients scheduled for elective surgery. The study aimed to recruit 90 participants but faced challenges due to the COVID-19 pandemic [72]. Participants were randomized into intervention and control groups, with the intervention group receiving standard care along with a VR-based perioperative journey experience. Results showed significant reductions in preoperative anxiety and stress levels among the intervention group compared to the control group, along with higher post-surgery satisfaction levels [72].

Cheng et al. conducted a quasi-experimental trial to assess the effectiveness of a combined intervention in reducing perceived stress and enhancing happiness, sleep quality, meditation experience, and life satisfaction among institutionalized older adults in Taiwan [73]. The results revealed significant improvements in happiness, perceived stress, sleep quality, meditation experience, and life satisfaction in the experimental group compared to the control group [73]. Gaggioli et al. conducted a multicentric randomized block-controlled trial involving teachers and nurses highly exposed to psychological stress to evaluate the efficacy of stress management interventions [74]. Participants were randomly assigned to an Experimental Group (EG), Control Group (CG), or Wait-List group (WL). The EG received stress management training using immersive VR scenarios, biosensors, and mobile phone applications, while the CG received traditional cognitive behavioural techniques without technology. Results showed significant reductions in chronic anxiety and perceived stress in both treatment groups, with greater improvements in the EG [74].

Dings et al. conducted a non-randomized controlled trial to investigate the effectiveness of two-dimensional (2D) video glasses and VR glasses in reducing pain and anxiety during vasectomy procedures [75]. A total of 176 patients scheduled for vasectomy were sequentially divided into three groups: Control, 2D video glasses, and VR glasses. Patients in the VR group experienced significantly higher levels of anxiety during the procedure compared to the control and 2D video glasses groups. Additionally, patients without prior hospitalizations reported higher levels of pain compared to those with prior hospitalizations. The study suggests that while 2D video glasses did not reduce pain or anxiety, VR glasses may increase anxiety levels during vasectomy procedures [75].

Discussion

In this study, we systematically reviewed and evaluated studies that assessed the effect of VR for stress management The purpose of this systematic review was to comprehensively examine the existing body of literature on the use of VR for stress management and contribute to the understanding of VR’s potential role as a viable and efficacious tool for stress reduction. The results of the included studies suggest that VRE is an effective tool for stress management. Stress is a highly prevalent issue in today’s society and is a significant contributor to the deterioration of mental well-being and human capital [76]. Individuals today frequently experience forms of stress rooted in various domains of their lives, and the inability to manage such stress can potentially result in a chronic imbalance of the sympathetic nervous system as well as mental challenges or stress-related disorders [2,77]. As described by the stress-vulnerability model, the outcome of stressful situations is dependent on the intricate interplay between the experienced stress level and the individual’s ability to cope or their vulnerability [8]. VR appears to be a generally low-risk intervention, as evidenced by the studies included in our review. While some participants may experience mild discomfort or motion sickness during VR sessions, serious adverse effects are rare. Additionally, the controlled nature of VR environments allows for careful monitoring and adjustment of stimuli to ensure participant safety.

Several studies have delved into the intricate relationship between VR and stress management within the realm of psychiatric disorders. While the results are diverse, they underscore the potential of VR as a therapeutic tool. Notably, when it comes to PTSD, VRET has yielded mixed outcomes. However, it is important to consider that the effectiveness of VR may vary depending on the specific trauma and individual responses [78] This variability highlights the need for personalized treatment approaches and further research to optimize VR-based interventions for PTSD. In addition, when paired with therapeutic techniques such as cognitive therapy, VR has demonstrated the ability to decrease psychiatric symptoms in participants experiencing psychosis or schizophrenia such as auditory verbal hallucinations, delusional conviction, distress, and depressive symptoms, while improving quality of life [30]. Furthermore, in the realm of phobias and anxiety disorders, VR has showcased its ability to alleviate symptoms, particularly in scenarios such as fear of public speaking and acrophobia. These findings suggest that VR can offer a versatile platform for ET in treating various anxiety-related conditions. Nonetheless, it is essential to address challenges such as fear renewal during virtual exposure, emphasizing the importance of refining VR protocols to maximize their benefits. In the context of stress-related disorders, the comparison between VR applications and traditional cognitive behavioural therapy unveils the advantages of VR, particularly in terms of accessibility and engagement. This is crucial because individuals with mental health issues often struggle with the initiative and energy required for conventional stress-reduction methods [79]. The user-friendly, low-effort nature of VR may bridge this gap and provide a more accessible route to stress management for those in need and can reduce the inconsistency of treatment delivery [80]. VR technology holds promise in the domain of stress management for psychiatric disorders, but its effectiveness depends on the specific condition and individual characteristics. Ongoing research and the refinement of VR-based interventions are essential to harness the full potential of this innovative approach to improving mental health.

The results regarding the application of VR in healthcare show that this is a dynamic and promising avenue of research. VR technology offers a wide array of possibilities that have already shown significant progress in enhancing patient care and overall well-being. Notably, VR has shown to be a valuable tool in pain management, with studies consistently demonstrating its effectiveness in reducing reported pain levels through interactive experiences and GM. This approach presents an alternative to traditional pain medications, engaging patients in a more holistic manner by providing distraction-based pain relief. In the realm of cancer care, VR has made substantial strides, particularly in mitigating anxiety, depression, and fatigue among chemotherapy patients, contributing significantly to their overall quality of life and psychological well-being [42,43]. This comprehensive approach to patient care acknowledges the emotional and psychological aspects of healthcare, extending beyond the purely physical. Beyond these areas, VR has found applications in various healthcare contexts, such as reducing anxiety related to medical procedures and improving rehabilitation programmes across diverse health conditions. During the COVID-19 pandemic, people were experiencing an influx of uncertainty, anxiety, and fear which can increase the risk of developing psychological distress and other mental health problems [77]. VR played a role in supporting mental health, with VR-based self-help interventions effectively reducing distress, stress, and depression during times of heightened uncertainty and isolation. VR's diverse applications in healthcare highlight its potential to improve patient care across various domains, and as technology continues to advance, becomes more affordable, and research expands, VR is poised to become an increasingly essential tool in enhancing healthcare outcomes and patient experiences.

Studies examining VR and work-related stress pose an interesting consideration regarding the effectiveness of VR as a stress-reduction and practice or capacity-building tool. These studies demonstrate how VR can benefit patients and practitioners alike. VR studies in relation to work stress have uncovered the ability to reduce mental demand and effort dimensions, emotional exhaustion, anxiety, and distress among varying types of medical residents as well as factory workers [68,69]. Each study reviewed in this section supports VR as a means to manage work-related stress, whether through a practice/training model or as a form of mindfulness and relaxation. In these instances, the VR intervention produced favourable results in comparison to control groups. These results further demonstrate the different potential approaches that can be taken to reduce stress and emphasize the therapeutic techniques that may be best suited to targeting specific stress-related behaviours or stress-inducing scenarios.

It's essential to consider the varying levels of stress experienced by different populations and how VR interventions may affect them differently. While our review primarily focuses on the efficacy of VR interventions in reducing stress across diverse populations and contexts, it's notable to compare the effectiveness observed in healthy participants facing daily stressors versus clinical populations experiencing more severe stress. Several studies included in our review investigated the impact of VR interventions on stress outcomes in healthy individuals facing everyday stressors. These studies consistently reported significant reductions in stress levels, indicating the potential of VR to effectively alleviate stress in this population. On the other hand, studies involving clinical populations facing more severe stress, such as patients with chronic autoimmune disorders or individuals with psychiatric disorders, also reported promising results. While both healthy participants and clinical populations may benefit from VR interventions in reducing stress, it's essential to recognize the differing levels and sources of stress experienced by these populations. Future research could further explore how VR interventions can be tailored to address the unique stressors faced by clinical populations, potentially enhancing their effectiveness in managing stress in these individuals. Furthermore, VR interventions present a versatile array of settings and durations, tailored to address specific populations and conditions. By offering immersive experiences that simulate real-life scenarios, VR enhances the effectiveness of stress management techniques. Moreover, when integrated with other therapeutic modalities such as cognitive behavioural therapy or GM, VR interventions may yield synergistic benefits. Their capacity to deliver personalized experiences and real-time feedback fosters active engagement and self-awareness among users. Additionally, VR-based interventions can be seamlessly adapted for use across various healthcare settings, from hospitals to rehabilitation centres. However, potential limitations and disadvantages must be considered, including the need for specialized equipment and trained personnel, variability in individual response to VR, and suitability concerns for certain medical conditions or sensory impairments. Addressing issues related to privacy, data security, and potential adverse effects of prolonged VR use is essential for the widespread adoption and ethical implementation of VR interventions.

In our analysis, we conducted a thorough assessment of the risk of bias across the included studies, and the findings revealed a generally low level of bias. This indicates that the studies we reviewed maintained a high standard of methodological rigour, minimizing the potential for systematic errors or inconsistencies in their results. This low risk of bias enhances the confidence in the robustness and credibility of the conclusions drawn from our analysis, providing a solid foundation for the assessment of the efficacy of VR interventions in managing stress outcomes across diverse populations and contexts. Additionally, considering the distribution of samples, we observed a diverse representation across different demographic groups, including individuals from various geographical regions and genders. However, it's essential to acknowledge the potential biases that may exist, such as the overrepresentation of certain demographic groups or cultural backgrounds, commonly referred to as WEIRD (Western, Educated, Industrialized, Rich, and Democratic) biases, and gender biases.

While our review encompasses a diverse range of populations and contexts, it's essential to acknowledge the limitations. The varied contexts in which VR has been studied for stress reduction suggest that its effectiveness may differ across domains, as evidenced by the contrasting results highlighted in our review. However, these discrepancies also present an opportunity for future research to delve deeper into unique contexts, expanding the current understanding of VR's potential in stress reduction. Moving forward, researchers should focus on identifying key factors that optimize VR-based interventions, such as determining the ideal exposure time and number of sessions required for desired outcomes. These insights are crucial for the development of more effective VR interventions in the future and should be a priority in prospective research and intervention trials. Additionally, our review has further limitations that require consideration. Firstly, there is the potential for publication bias, as we restricted our analysis to studies published in peer-reviewed journals, possibly overlooking relevant unpublished. Secondly, the variability in study quality across the included literature, with some studies lacking rigorous experimental designs or adequate control groups, introduces the possibility of bias and may impact the reliability of our conclusions. Furthermore, despite our efforts to conduct a comprehensive search across multiple databases, it is possible that relevant studies were missed, potentially limiting the scope of our review. Small number of studies, small sample size and diversity of participants is another limitation. Additionally, the exclusion of non-English language studies may have introduced language bias. Moreover, our decision not to conduct a meta-analysis due to methodological heterogeneity among the included studies means that we were unable to quantitatively assess the overall effect size of VR interventions on stress outcomes. Finally, given the rapidly evolving nature of VR technology and its applications for stress management, our review may not encompass the most recent advancements in the field. Further exploration into the long-term effectiveness of VR interventions is vital to strengthen findings. Understanding how VR integrates into clinical practice and tailoring interventions to diverse populations is crucial.

## Conclusions

In conclusion, VR-based interventions maintain the ability to become extremely personalized to the user, creating a patient-centred care model that can adapt and flux along with the individual’s needs. Centring care around the patient and engaging them in their care has been associated with better health outcomes and quality of life. VR allows for control, support and customization throughout the therapeutic process that may not be otherwise available, especially regarding instances such as exposure therapy, where controlling the stimuli may be impossible if not for VR technology and programming. Reports of positive participant perceptions of VR, high feasibility of VR intervention implementation, and high participant retention frequently accompanied studies implementing VR-based interventions. In cases where analysis found no notable changes in participants or differences from the control groups, researchers still appear to agree that even without statistically significant results, VR-based interventions did not cause aversive outcomes in participants. Given this information, it might be concluded that VR acts as a low-risk intervention for stress management for a wide-ranging patient demographic.

## Appendices

**Table 2:** Risk of bias assessment for randomized studies-Cochrane Handbook.

Author	Bias Arising from the Randomization Process	Bias Due to Deviations from Intended Intervention	Bias Due to Missing Outcome Data	Bias in the Measurement of the Outcome	Bias in Selection of the Reported Result
Reger et al. 2019 [26]	Low	Low	Some concerns	Low	Low
Loucks et al. 2019 [28]	Low	Low	Low	Low	Low
Vieira et al. 2018 [48]	Low	Low	Low	Low	Low
Freeman et al. 2016 [30]	Low	Low	Low	Low	Low
Gullo et al. 2023 [43]	Low	Low	Some concerns	Low	Low
Farahimanesh et al. 2023 [63]	Low	Low	Low	Low	Low
Fabi et al. 2022 [42]	Low	Some concerns	Low	Low	Low
Zhang et al. 2022 [37]	Low	Low	Low	Low	Low
Aganov et al. 2022 [36]	Low	Low	Low	Low	Low
Kim et al. 2022 [29]	Low	Low	Some concerns	Low	Low
Vlake et al. 2022 [65]	Low	Some concerns	Low	Low	Low
Rutkowski et al. 2021 [49]	Low	Low	Low	Low	Low
Lewandowski et al. 2021 [51]	Low	Low	Low	Low	Low
Weitzman et al. 2021 [69]	Low	Low	Low	Low	Low
Richesin et al. 2021 [32]	Low	Some concerns	Low	Low	Low
Brown et al. 2020 [46]	Low	Low	Low	Low	Low
Chirico et al. 2020 [41]	Low	Low	Some concerns	Low	Low
Venuturupalli et al. 2019 [61]	Low	Low	Low	Low	Low
Kothgassner et al. 2019 [33]	Low	Low	Low	Low	Low
Villani et al. 2012 [34]	Low	Low	Low	Low	Low
Jóźwik et al. 2022 [50]	Low	Low	Low	Low	Low
Almedhesh et al. 2022 [47]	Low	Some concerns	Low	Low	Low
Tan et al. 2021 [35]	Low	Low	Low	Low	Low
Yang et al. 2019 [44]	Low	Some concerns	Low	Low	Low
Vaquero-Blasco et al. 2021 [71]	Low	Low	Low	Low	Low
Chiu et al. 2023 [72]	Low	Low	Low	Some concerns	Low
Zolfaghari et al. 2022 [53]	Low	Low	Low	Low	Low
Dings et al. 2021 [75]	Low	Low	Low	Low	Low
Szczepanska-Gieracha et al. 2021 [57]	Low	Low	Low	Low	Low
Wang et al. 2020 [55]	Low	Some concerns	Low	Low	Low
Gaggioli et al., 2014 [74]	Low	Low	Low	Low	Low
Veling et al. 2021 [58]	Low	Low	Low	Low	Low
Pallavicini et al. 2009 [60]	Low	Some concerns	Low	Low	Low
Blum et al. 2009 [59]	Low	Low	Low	Low	Low

**Table 3:** Risk of bias assessment of non-randomized studies.

Author	Selection	Comparability	Outcome	Total score
Afifi et al. 2023 [56]	3	2	2	7
O’Gara et al. 2022 [70]	2	3	2	6
Riva et al. 2021 [64]	2	2	3	7
Anderson et al. 2017 [52]	3	2	3	8
Croghan et al. 2022 [67]	3	3	3	9
Hoch et al. 2012 [31]	2	2	2	6
Schneider et al. 2007 [40]	2	2	3	7
Schneider et al. 2003 [39]	2	2	3	7
Meyer et al. 2022 [62]	3	2	3	8
Shah et al. 2015 [54]	2	3	3	8
Cheng et al. 2020 [73]	3	3	3	9
Ganry et al. 2018 [45]	3	3	3	9
Espinoza et al. 2012 [38]	2	3	3	8
Ho et al. 2023 [68]	3	3	3	9
Beverly et al. 2022 [66]	3	3	3	9
